# DOES EXPOSURE TO INFORMATION ABOUT DEMENTIA CHANGE STIGMA? AN EXPERIMENTAL STUDY

**DOI:** 10.1093/geroni/igz038.3541

**Published:** 2019-11-08

**Authors:** Fan Zhang, Sheung-Tak Cheng

**Affiliations:** The Education University of Hong Kong, Hong Kong, Hong Kong

## Abstract

Objectives: Educational programs on dementia may backfire, as recipients could feel more negatively about people with dementia after exposure to the alarming symptoms (e.g., behavioral and psychological symptoms of dementia, or BPSD). This study aimed to investigate whether such exposures had any effect on stigma. Methods: 200 adults aged 18-83 years were randomly assigned to three groups. The first group read vignettes describing fictitious older adults with memory impairment. The second group read the same vignettes that were expanded to include descriptions of BPSD (i.e., memory impairment cum BPSD). After reading the vignettes, both groups answered questions about stigma, while the third group directly responded to this questionnaire without reading any vignette (i.e., not exposed to experimental manipulation). ANOVA was performed to analyze the effect of experimental manipulation, as well as that of age, education, whether having relatives with dementia, and belief about treatability of dementia. Results: At posttest, the level of stigma was moderate and was comparable across the three groups, suggesting that exposures to information about cognitive and behavioral symptoms did not change people’s stigmatizing attitude. The absence of group effect in stigma did not vary by age, education, whether having a relative with dementia, or belief about prognosis. Only the main effects of age and education were significant, where younger and least educated participants reported higher stigma. Conclusion: There was no evidence that stigma would be affected by exposure to information about symptoms of dementia, including the more disturbed ones (i.e., BPSD).

